# Evaluation of Alginate Hydrogel Microstrands for Stromal Cell Encapsulation and Maintenance

**DOI:** 10.3390/bioengineering11040375

**Published:** 2024-04-13

**Authors:** Sujith Chander Reddy Kollampally, Xulang Zhang, Nicholas Moskwa, Deirdre A. Nelson, Susan T. Sharfstein, Melinda Larsen, Yubing Xie

**Affiliations:** 1 Department of Nanoscale Science and Engineering, College of Nanotechnology, Science, and Engineering, University at Albany, State University of New York, 257 Fuller Road, Albany, NY 12203, USA; skollampally@albany.edu (S.C.R.K.); xzhang45@albany.edu (X.Z.); ssharfstein@albany.edu (S.T.S.); 2 Department of Biological Sciences and The RNA Institute, University at Albany, State University of New York, 1400 Washington Ave., Albany, NY 12222, USA; Nicholas.Moskwa@jax.org (N.M.); dnelson@albany.edu (D.A.N.); mlarsen@albany.edu (M.L.); 3 The Jackson Laboratory of Genomic Medicine, 10 Discovery Drive, Farmington, CT 06032, USA

**Keywords:** alginate, hydrogel, microfiber, mesenchymal stromal cell, encapsulation, cell delivery, cell therapy, fibrotic

## Abstract

Mesenchymal stromal cells (MSCs) have displayed potential in regenerating organ function due to their anti-fibrotic, anti-inflammatory, and regenerative properties. However, there is a need for delivery systems to enhance MSC retention while maintaining their anti-fibrotic characteristics. This study investigates the feasibility of using alginate hydrogel microstrands as a cell delivery vehicle to maintain MSC viability and phenotype. To accommodate cell implantation needs, we invented a Syringe-in-Syringe approach to reproducibly fabricate microstrands in small numbers with a diameter of around 200 µm and a porous structure, which would allow for transporting nutrients to cells by diffusion. Using murine NIH 3T3 fibroblasts and primary embryonic 16 (E16) salivary mesenchyme cells as primary stromal cell models, we assessed cell viability, growth, and expression of mesenchymal and fibrotic markers in microstrands. Cell viability remained higher than 90% for both cell types. To determine cell number within the microstrands prior to in vivo implantation, we have further optimized the alamarBlue assay to measure viable cell growth in microstrands. We have shown the effect of initial cell seeding density and culture period on cell viability and growth to accommodate future stromal cell delivery and implantation. Additionally, we confirmed homeostatic phenotype maintenance for E16 mesenchyme cells in microstrands.

## 1. Introduction

MSCs are multipotent stem cells that can be isolated from various tissues, including bone marrow [1], adipose tissue [2], and umbilical cord [3], and are of interest for their regenerative responses in vivo. Apart from their multilineage differentiation capacity [1,4,5], MSCs have been shown to possess immunomodulatory [6,7,8,9] and anti-inflammatory properties [10,11,12], the capability of secreting bioactive molecules for tissue repair [13,14], and the ability to modulate fibrotic responses [15]. These diverse characteristics make MSC-based cell delivery of interest for therapeutics for bone, cartilage, ligament, skin, heart, and other organs. Since MSCs can be home to and target inflamed tissue [16,17,18,19], their survival, retention, and engraftment play a crucial role in their utilization as therapeutic agents, including localized and targeted therapies [20,21,22]. However, transplanted MSCs face many shortcomings, one of which is low MSC survival, retention, and homing efficiency post-delivery due to undesired biodistribution in a host, for example, being entrapped by the lung [23,24,25,26,27]. This challenge highlights the need for delivery vehicles that can improve the survival and local retention of MSCs, which may enhance their regenerative and anti-inflammatory/fibrotic properties [28]. An optimal delivery vehicle should maximize the regenerative efficacy while maintaining the viability, phenotype, and functionality of MSCs. Hydrogels have shown feasibility for delivering MSCs in vivo and demonstrated improved MSC survival and retention [29,30,31].

In particular, alginate hydrogels can support the delivery of MSCs both in ex vivo and in vivo settings [32,33,34]. Alginate is a naturally occurring anionic polysaccharide heteropolymer derived from brown seaweed, consisting of alternating blocks of (1,4)-linked β-D-mannuronate (M) and its C5-epimer, α-L-guluronate (G) [35]. The composition of these blocks influences the physical and chemical properties of alginate [35,36]. Alginate’s ability to form hydrogels in the presence of divalent cations, such as Ca^2+^ and Ba^2+^, is attributed to the carboxylate groups present in its structure [37,38]. The crosslinking network of alginate hydrogels enables them to simulate ECM-like characteristics such as high water content, porosity, permeability, and viscoelasticity [36,38,39,40,41,42]. The advantages of alginate hydrogels, including ease of chemical modification (addition of bioactive cues [43,44,45,46,47,48,49] and therapeutic agents), support of cell viability (biocompatibility [43,50], mild gelation, and soft-tissue-like mechanical properties), and ease of transplantation (biodegradability [32], malleability [39,40], and high ionic conductivity [51]), have made it a popular biomaterial for drug delivery systems [52], tissue engineering scaffolds [33], and encapsulation of living cells [39].

Alginate hydrogels have been explored in different structures for MSC delivery, such as discs or sheet-like gels [32,53,54,55,56], microbeads [57], microfibers [58,59], and 3D-printed geometries [60], showing enhanced cell retention and implanted cell survival [61]. Among these alginate hydrogels, microtubular structures with diameters that range from 20 to 800 μm (e.g., microtubes, microfibers, microribbons, microstrands) have been used for long-term cultures, which maintain good cell viability and show great potential to support cell proliferation and function in vitro and in vivo [39,40]. The high porosity of tubular hydrogels offers a large surface area and space for cellular organization, proliferation, and expansion in vitro [62]. In this study, we sought to use 3D, cell-laden alginate hydrogel microstrands with long, thin, fiber-like structures featuring diameters that range from 100 to 300 μm. The thin fiber structure facilitates efficient radial diffusion of nutrients and oxygen across the microstrands for cell growth and differentiation. The long fiber structure facilitates the handling and retrieving of the cell-laden microstrands, enabling improved cell delivery of MSCs for tissue regeneration studies [39,40]. Current approaches to fabricating these cell-laden hydrogel microstrands, microfibers, and core-shell microtubes include microfluidics [39,63,64], wet spinning [65], extrusion [66,67,68], and 3D printing [69]. However, it is challenging to use these methods to generate small volumes of microstrands with high cell densities for cell implantation in small animal models.

To address these challenges, we developed a Syringe-in-Syringe (SiS) device to fabricate alginate hydrogel microstrands in small volumes suitable for cell implantation in mouse models. We optimized the assembly of the microstrands containing stromal cells and determined the feasibility of using alginate hydrogels to support MSC growth and viability and maintain their anti-fibrotic properties. Using NIH 3T3 cells, we evaluated cell encapsulation and recovery efficiency and the effect of initial cell seeding density on cell viability and growth in microstrands. Due to their MSC-like properties and their ability to support early differentiation of salivary gland epithelium, we also examined survival and phenotypic maintenance for primary embryonic 16 (E16) salivary mesenchyme cells in the microstrands.

## 2. Materials and Methods

### 2.1. Preparation of Hydrogel Solutions and Viscosity Measurement

Sodium alginate (FUJIFILM Wako Pure Chemicals Co., Osaka, Japan) was dissolved in 0.9% sodium chloride solution (NaCl, Sigma-Aldrich, St Louis, MO, USA) at a concentration of 1.5% *w*/*v* or 3% *w*/*v* and then autoclaved at 121 °C for 15 min. The sterilized solutions were stored at 4 °C for future use. The 100 mM CaCl_2_ (Sigma-Aldrich) solution based on previous optimization studies [67] was prepared and autoclaved at 121 °C for 15 min and stored at room temperature for future use.

The viscosity of the alginate solution was measured using a BS/U capillary Ostwald viscometer (DC Scientific, Glen Burnie, MD, USA). Briefly, 1.5% *w*/*v* alginate solution was injected into the U-shaped Ostwald viscometer, and the time required to pass through the bulb due to the capillary hydrostatic pressure was measured. The kinematic viscosity (*ν*) (mm^2^/s) was determined by multiplying the constant of the viscometer (*C* = 0.0272) and the transit time (*t*), as shown in Equation (1):

*ν* = *C* × *t* (1)

### 2.2. Construction of Syringe-in-Syringe (SiS) Device for Fabrication of Alginate Hydrogel Microstrands

**Figure 1:**
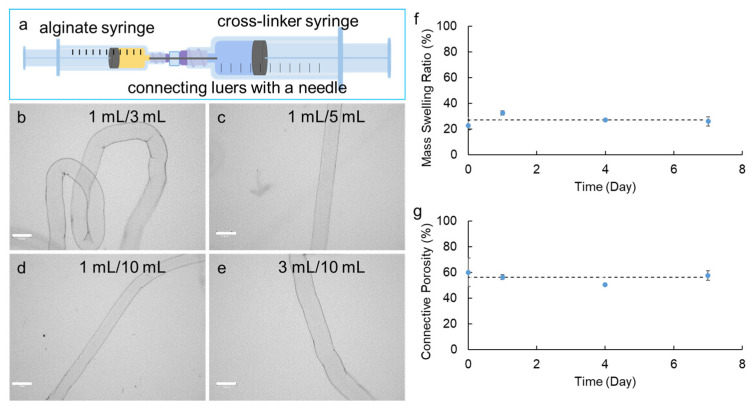
Fabrication of alginate hydrogel microstrands using the Syringe-in-Syringe (SiS) approach. (**a**) Representative SiS device. (**b**–**e**) Optical images of alginate hydrogel microstrands fabricated by SiS device with alginate syringe volume/capacity to cross-linker syringe volume/capacity of 1 mL/3 mL (**b**), 1 mL/5 mL (**c**), 1 mL/10 mL (**d**), and 3 mL/10 mL (**e**). Scale bar = 300 µm. (**f**) Mass swelling ratio and (**g**) connective porosity of alginate hydrogel microstrands fabricated by the 1 mL/5 mL Syringe-in-Syringe device when incubated with DMEM for 7 days. The dashed line shows the average swelling ratio on days 0, 1, 4, and 7.

To fabricate alginate hydrogel microstrands suitable for cell implantation studies, we constructed a Syringe-in-Syringe device (SiS) (Figure 1a). The SiS device contains two syringes (BD, Franklin Lakes, NJ, USA) of either the same volume or different volumes. A needle (blunt end) (Hamilton Company, Reno, NV, USA) with plastic or steel luer locks was used to bridge the two syringes with the aid of adapters (Cole Parmer, Marysville, WA, USA). An alginate solution of 1.5% *w*/*v* was loaded into one syringe (alginate syringe), while the other syringe (cross-linker syringe) was loaded with 100 mM CaCl_2_. The plunger on the cross-linker syringe was then pulled, creating a negative pressure along the needle bridge to draw the alginate solution from the alginate syringe into the cross-linker syringe. As the alginate solution encountered the calcium ions in the cross-linker syringe, immediate crosslinking occurred to form alginate hydrogel microstrands. Different syringe volume/capacity combinations of SiS devices were constructed, including 1 mL alginate syringe/1 mL cross-linker syringe, 1 mL/3 mL, 1 mL/5 mL, 1 mL/10 mL, 3 mL/3 mL, 3 mL/5 mL, 3 mL/10 mL, 5 mL/5 mL, and 5 mL/10 mL, connected with a blunt end needle (30G). The volume of 1.5% *w*/*v* alginate solution in the alginate syringe was kept constant (250 µL), and the volume of 100 mM CaCl_2_ solution was approximately 70% of the cross-linker syringe capacity (e.g., 0.7 mL CaCl_2_ for 1 mL cross-linker syringe, 2.3 mL CaCl_2_ for 3 mL syringe, etc.). The feasible and optimal conditions to fabricate 250 µL alginate hydrogel microstrands with a diameter of around 200 µm were investigated.

### 2.3. Connective Porosity and Mass Swelling Ratio of Alginate Hydrogel Microstrands

To characterize the physical properties of alginate hydrogel microstrands, the swelling ratio and porosity of these microstrands were evaluated based on previously reported methods [70,71] immediately after fabrication (day 0) or after incubation in DMEM for 1, 4, or 7 days. In each pre-weighted 35-mm dish (*W*_1_), the alginate hydrogel microstrand sample was added, aspirating away any trace amounts of water around the microstrand. The weight of the dish with the microtube inside was recorded as *W*_2_. The mass of the swollen hydrogel microstrand (*M_Wet_*) was calculated by subtracting *W*_1_ from *W*_2_.

To determine the connective porosity, all the water within the interconnected pores of the alginate hydrogel microstrand was removed with a Kim wipe, followed by measuring the weight of the dish with the microstrand (*W*_3_). The weight of the microstrand after the liquid was wicked away from connective pores was calculated by subtracting *W*_3_ from *W*_1_. Therefore, the weight of liquid wicked away from connective pores equaled to subtracting (*W*_3_ − *W*_1_) from (*W*_2_ − *W*_1_), i.e., (*W*_2_ − *W*_3_). Assuming the density of DMEM is close to the water density of 1 g/cm^3^ (i.e., 1 g/mL) [72], the volume of connective pores (*V_Pore_*) was calculated by Equation (2):

(2) $$V_{Pore}=\frac{(W_{2}-W_{3})\;g}{1\;g/\mathrm{mL}}$$

Similarly, assuming the density of an alginate hydrogel is around 1 g/mL [73], the volume of the microstrand (*V_Microstrand_*) was calculated by Equation (3):

(3) $$V_{Microstrand}=\frac{(W_{2\;}-W_{1})\;g}{1\;g/\mathrm{mL}}$$

The connective porosity (%) was calculated by Equation (4):

(4) $$Connective\;Porosity\;(\%)=\frac{V_{pore}}{V_{Microstrand\;}}\times 100\%$$

To determine the mass swelling ratio, the alginate hydrogel microstrand was further air-dried in the pre-weighed dish (*W*_1_) overnight. The weight of the dish with the air-dried microstrand was recorded as *W*_4_. The mass of the dried microstrand (*M_dry_*) was calculated by subtracting *W*_1_ from *W*_4_. The mass swelling ratio was calculated by Equation (5):

(5) $$Mass\;Swelling\;Ratio=\frac{M_{Wet\;}}{M_{Dry\;}}\times 100\%$$

### 2.4. Cell Culture

#### 2.4.1. Culture of Murine NIH 3T3 Fibroblasts

Murine NIH 3T3 fibroblasts were maintained in high glucose Dulbecco’s Modified Eagle’s Medium (DMEM) (Sigma-Aldrich), supplemented with 10% fetal bovine serum (FBS) (Sigma-Aldrich) and 1% penicillin–streptomycin (pen/strep, 10,000 units penicillin/10 mg streptomycin from Sigma-Aldrich), in a 37 °C, 5% CO_2_ humidified incubator. The medium was changed one day before the subculture, and the subculture was performed every two to three days.

#### 2.4.2. Isolation and Culture of Murine Primary E16 Salivary Mesenchyme Cells

Murine primary E16 mesenchyme cells were isolated from salivary glands of embryonic day 16 (E16) timed-pregnant CD-1 female mice from Charles River Laboratories (Wilmington, MA, USA), as previously described [74,75,76]. The care and handling of mice was carried out in accordance with the National Institutes of Health Guide for the Care and Use of Laboratory Animals and with protocols approved by the Institutional Animal Care and Use Committee (IACUC) of the University at Albany, State University of New York. Primary E16 mesenchyme cells were cultured in DMEM/F12 (Sigma-Aldrich), supplemented with 10% FBS and 1% pen/strep for 4 days in a 37 °C, 5% CO_2_ humidified incubator. The medium was changed on day 1 and day 3.

### 2.5. Encapsulation of Cells in Alginate Hydrogel Microstrands Using the Syringe-in-Syringe (SiS) Device

Murine NIH 3T3 fibroblasts were used as a stromal cell model to explore the feasibility of generating 250 µL of alginate hydrogel microstrands for cell encapsulation and future implantation studies, and to determine encapsulation, recovery efficiency, cell viability, and growth in these microstrands. Briefly, 125 μL of 3% *w*/*v* sterile alginate solution was mixed well with 125 μL of cell culture medium (DMEM + 10% FBS + 1% pen/strep) to yield a final alginate concentration of 1.5%. NIH 3T3 fibroblasts were trypsinized with 0.05% trypsin/EDTA (Sigma Aldrich) and neutralized with FBS in a cell culture medium. Cells of 0.25, 0.5, 1, 2, or 5 × 10^6^ were aliquoted into 15 mL sterile centrifuge tubes, respectively, and centrifuged at 1200 rpm for 5 min. After carefully removing the supernatant, 250 μL of 1.5% alginate-medium solution was added to the cell pellets and gently pipetted up and down to ensure uniform mixing of cells with the alginate-medium solution, resulting in initial cell seeding densities of 1, 2, 4, 8, and 20 × 10^6^ cells/mL alginate solution, respectively. The cell–alginate solution was then loaded into a sterile 1 mL BD syringe, i.e., the alginate syringe. Then, 100 mM CaCl_2_ solution was loaded into a 5 mL BD syringe, i.e., the cross-linker syringe. Both syringes were then joined using an adapter with a blunt-ended needle (30 G) that bridged both solutions in each syringe. The plunger on the cross-linker syringe was then pulled, creating a negative pressure to draw the cell–alginate mixture from the alginate syringe into the CaCl_2_ solution in the cross-linker syringe to form cell-laden alginate hydrogel microstrands. The cell-laden hydrogel microstrands were maintained in the CaCl_2_ solution for 10–20 s and then transferred to a 70 µm Falcon cell strainer (Thermo Fisher Scientific, Pittsburgh, PA, USA), followed by immersion in 0.9% NaCl solution to remove excess CaCl_2_. The cell strainer containing cell-laden hydrogel microstrands was then transferred to a six-well plate with 6 mL of media for NIH 3T3 fibroblasts (DMEM + 10% FBS + 1% pen/strep) supplemented with 25 mM CaCl_2_ to maintain the hydrogel integrity during cell culture. Six mL of medium was added to each well to ensure that the microstrands in the cell strainer were fully immersed in the medium. The microstrands were evenly distributed in the cell strainer fitted in the six-well plates, allowing for proper diffusion of oxygen and nutrients into the microstrands. The medium was changed daily, leaving 0.5 mL conditioned medium and adding 5.5 mL fresh medium to allow for cell growth for 4 days.

Primary E16 salivary mesenchyme cells were also encapsulated in alginate hydrogel microstrands in the same manner as the NIH 3T3 cells, except that E16 mesenchyme cell pellets contained 2.5 × 10^5^, 5 × 10^5^, and 1 × 10^6^ cells, respectively. E16 mesenchyme cell-laden hydrogel microstrands at initial cell seeding densities of 1, 2, and 4 × 10^6^ cells/mL alginate solution were prepared as described above, transferred in a cell strainer fitted in a six-well plate, and cultured in 6 mL of media (DMEM/F12 + 10% FBS + 1% pen/strep) supplemented with 25 mM CaCl_2_ for 3 days to evaluate cell viability and growth and for 4 days to evaluate mesenchymal marker expression.

### 2.6. Optical Imaging of Alginate Hydrogel Microstrands

Optical images of microstrands were taken using an EVOS M7000 Imaging System (Themo Fisher Scientific, Waltham, MA, USA). The diameter of the hydrogel microstrand was measured using Celleste 6 image analysis software 6 and calculated as the mean ± standard deviation. Optical images of cell growth in alginate hydrogel microstrands were also acquired using an EVOS M7000 Imaging System on days 0, 1, 3, and 4.

### 2.7. Trypan Blue Exclusion Assay

The trypan blue exclusion assay was used to determine cell viability before cell encapsulation in alginate hydrogel microstrands and after cell culture in the microstrands. At the conclusion of each cell culture period, the cell-laden microstrands, which were placed on a cell strainer in a six-well plate, were first removed from their cell media and then rinsed with 0.9% NaCl solution. Subsequently, the microstrands were transferred to a new six-well plate. To dissolve the microstrands, 1–2 mL of 55 mM sodium citrate solution was added for 5–10 min, followed by adding 1–2 mL of fresh cell culture media. For the trypan blue staining, equal volumes of the cell suspension and 0.40% Trypan Blue Dye (BIO-RAD, Hercules, CA, USA) were mixed by gently pipetting up and down 10 times in a sterile microcentrifuge tube. Then, 10 µL of this mixture was loaded to each side of a Brightline hemocytometer (Sigma-Aldrich), allowing for the counting of unstained viable cells and blue-stained dead cells in the four outer grids using a manual cell counter.

### 2.8. LIVE/DEAD Cell Assay

Cell viability in microstrands was further confirmed by Invitrogen LIVE/DEAD Viability/Cytotoxicity kit (Thermo Fisher Scientific) followed by fluorescence imaging using the EVOS M7000 Imaging System. On day 4, cell-laden microstrands in the cell strainer were incubated with 2 µM calcein AM and 4 µM ethidium homodimer-1 (EthD-1) in 6 mL of cell culture media at room temperature for 30 min. Microstrands were then transferred to a Platinum Line cover glass slide (25 × 75 mm) (Waldemar Knittel Glasbearbeitungs, Braunschweig, Germany) and observed with an EVOS M7000 Imaging System with an EVOS LIGHT CUBE, GFP 2.0 (excitation at 494 nm/emission at 540 nm) to visualize live cells that convert calcein AM to green fluorescent calcein and with an EVOS Light Cube, Texas Red 2.0 (excitation at 595 nm/emission at 613 nm) to visualize red fluorescent EthD-1-stained dead cells. To visualize 3D cell distribution and organization, a movie (.WMV) was created based on a 3D reconstruction of Z-stacked fluorescent images of LIVE/DEAD stained cells in alginate hydrogel microstrands using the Celleste 6 Image Analysis Software 6 with 3D Visualization Module (Thermo Fisher Scientific).

### 2.9. AlamarBlue Assay of Viable Cell Growth in Alginate Hydrogel Microstrands

To quantify NIH 3T3 fibroblasts and primary E16 mesenchyme cells in alginate hydrogel microstrands from different initial cell seeding densities over a period of time, an alamarBlue assay (Thermo Fisher Scientific) was performed. To optimize the alamarBlue assay for cells grown in alginate hydrogel microstrands, we first encapsulated 150,000, 300,000, 600,000, and 1,000,000 cells in 250 µL microstrands, respectively, and added 120 µL or 600 µL alamarBlue solution to 6 mL media. In both cases, the standard curve showed high linearity R^2^ > 0.99 (Figure S1). To determine cell growth in microstrands, we chose to add 120 µL alamarBlue solution to 6 mL media (2%) for the assay to save the reagent and, in particular, to avoid the potential cytotoxicity effect of the alamarBlue dye [77,78,79].

For NIH 3T3 fibroblasts, cells were encapsulated in 250 µL alginate hydrogel microstrands at initial cell seeding densities of 1, 2, 4, 8, and 20 × 10^6^ cells/mL alginate solution, respectively, and cultured in a humidified incubator at 37 °C, 5% CO_2_ for 3 h (day 0) and 1–4 days (day 1, day 2, day 3, and day 4). For primary E16 mesenchyme cells, cells were encapsulated in 250 µL alginate hydrogel microstrands at initial cell seeding densities of 1, 2, and 4 × 10^6^ cells/mL alginate solution, respectively, and cultured in a humidified incubator at 37 °C, 5% CO_2_ for 3 h (day 0) for 1–3 days (day 1 and day 3). To prepare the standard curve for each time point, 0.25, 0.5, 1, 2, and 5 × 10^6^ viable NIH 3T3 fibroblasts were encapsulated in 250 µL alginate hydrogel microstrands and incubated in media at 37 °C, 5% CO_2_ for 3 h.

To initiate the alamarBlue assay, 6 mL fresh medium was added to the microstrands present on the cell strainer fitted in each well of the six-well plate. Then, 120 µL of alamarBlue reagent was added to each well. After incubation for six hours, 100 µL of medium in triplicate was taken from the standard curve plates and sample plates, respectively, and loaded into the same 96-well plate. The plate was then read for fluorescence intensity in relative fluorescence units (RFU) at excitation/emission 545 nm/590 nm with negative controls (medium only and microstrands only) using Infinite® 200 PRO microplate reader (Tecan, Männedorf, Switzerland). The standard curve of fluorescence intensity (RFU) vs. viable cell number was plotted. Subsequently, a linear regression equation was obtained from each standard curve. The cell number in microstrands was calculated based on the standard curve. We then validated our alamarBlue assay for measuring cell number in high-density alginate hydrogel microstrands by plotting the measured cell number using alamarBlue assay vs. cell number from trypan blue cell counting, which showed good correlation with slope = 0.9926 and R^2^ = 0.9903 (Figure S2).

### 2.10. Immunocytochemistry Analysis

To evaluate the protein expression by cells in alginate hydrogel microstrands, immunocytochemistry was performed. Cell-laden hydrogel microstrands in cell strainers fitted in a six-well plate were rinsed with 0.9% NaCl and then fixed in 4% paraformaldehyde (Sigma-Aldrich) in 0.9% NaCl for 30 min on ice. Samples were then rinsed with 0.1% Tween 20 (Thermo Fisher Scientific) in 0.9% NaCl three times, rocking at 40 rpm for 5 min each time. The cell-laden microstrands were then permeabilized in 0.1% Triton X-100 (Sigma-Aldrich) in 0.9% NaCl rocking at 40 rpm for 15 min at room temperature. Three more wash steps were performed with 0.1% Tween 20 in 0.9% NaCl solution, followed by blocking with 20% MilliporeSigma donkey serum (Sigma-Aldrich, Burlington, MA, USA) in 0.9% NaCl for 1–2 h at room temperature. The primary antibody was diluted in 3% bovine serum albumin (BSA) (Thermo Fisher Scientific) in 0.9% NaCl and incubated with cell-laden microstrands overnight at 4 °C on a rocker. Antibodies used were anti-vimentin (1:400, V2258, Sigma-Aldrich), anti-PDGFRα (1:100, AF1062, R&D systems), and anti-α-SMA (1:400, A5228, Sigma-Aldrich). After washing with 0.1% Tween 20 in 0.9% NaCl four times with 10 min rocking per wash, the respective secondary antibodies, along with 4′,6-diamidino-2-phenylindole (DAPI) (1:400, Sigma-Aldrich), were added to each sample and incubated for 2 h on a rocker at room temperature (Alexa Fluor^®^ 647 donkey anti-mouse IgG (1:250, 715–606-150, Jackson ImmunoResearch Laboratories, West Grove, PA, USA for vimentin and α-SMA) or Alexa Fluor^®^ 488 donkey anti-goat IgG (1:250, 705-545-147, Jackson ImmunoResearch Laboratories for PDGFRα)), respectively. The microstrands were washed with 0.1% Tween 20 in 0.9% NaCl three more times before being mounted on slides with EMS Fluoro-Gel mounting media (1:100 PPD anti-fade solution, Thermo Fisher Scientific). Confocal microscopy was performed on a Leica Confocal Microscope TCS SP-5 controlled by LAS-AF software version 2.7.3.9723 using 10× and 63× (oil immersion) objectives (Leica Microsystems, Mannheim, Germany), with excitation/emission 499/520 nm for vimentin and α-SMA (Alexa488), 565/576 nm for PDGFRα (Alexa647), and 350/470 nm for DAPI.

The expression of vimentin, PDGFRα, and α-SMA of primary E16 mesenchyme grown in alginate hydrogel microstrands was further quantified as the percentage of cells expressing each marker (*N_Total nuclei_* − *N_Nuclei with no marker expression_*) among the total cell population (*N_Total nuclei_*) identified by DAPI-stained nuclei using Equation (6).

(6) $$\%\;Marker\;Expression=\frac{N_{Total\;nuclei}-N_{Nucei\;with\;no\;marker\;expression\;}}{N_{Total\;nuclei}}\times 100\%$$

### 2.11. Statistical Analysis

Data are presented as mean ± standard deviation and were analyzed by ordinary one-way analysis of variance (ANOVA) using GraphPad Prism 9.2.0 for comparison between different groups or between different days within the same initial cell seeding density group. *p* < 0.05 was considered to be statistically significant. Each experiment was repeated at least three times for NIH 3T3 fibroblasts and twice for primary E16 mesenchymal cells.

## 3. Results

### 3.1. Effect of the Syringe Volume/Capacity for Construction of Syringe-in-Syringe (SiS) Devices on the Diameter of Hydrogel Microstrands

To investigate the feasibility of using the SiS device to fabricate implantable alginate hydrogel microstrands with a diameter of around 200 µm for cell delivery and implantation studies, we explored different syringe combinations of SiS devices. Specifically, we focused on variations in the capacity of the alginate syringe (1 mL, 3 mL, and 5 mL) and cross-linker syringe (1 mL, 3 mL, 5 mL, and 10 mL) while keeping the needle size constant (30G, inner diameter 159 µm). While the actual volume of alginate solution (1.5% *w*/*v*, kinematic viscosity measured to be 24.5 ± 0.06 mm^2^/s at room temperature) loaded in each alginate syringe remained constant at 250 µL, we loaded each cross-linker syringe with 100 mM CaCl_2_ in the actual volume around 70% of its full capacity. Analyzing the diameters of the resulting microstrands generated from these SiS devices with different combinations allowed us to determine the feasibility of fabricating alginate hydrogel microstrands with various combinations and to assess the reproducibility of this fabrication method (Table 1).

Successful microstrand fabrication using SiS devices was feasible when the ratio of the alginate syringe volume/capacity to the cross-linker syringe volume/capacity was 1:3 or higher (i.e., 1 mL/3 mL, 1 mL/5 mL, 1 mL/10 mL, 3 mL/10 mL syringe combination) (Figure 1b–e). This ratio seemed to be essential for creating the necessary negative pressure to pull the alginate solution from the alginate syringe with the connecting needle into the CaCl_2_ solution in the cross-linker syringe to form the microstrands (Table 1). While keeping the needle size, the volume of alginate used, and the alginate syringe capacity constant, increasing the capacity of the cross-linker syringe led to a decrease in the diameter of microstrands. Additionally, increasing the capacity of the alginate syringe from 1 mL to 3 mL while keeping the cross-linker syringe capacity at 10 mL increased the diameter of microstrands. In particular, using 1 mL/3 mL, 1 mL/5 mL, 1 mL/10 mL, and 3 mL/10 mL SiS devices, we were able to repeatedly produce alginate hydrogel microstrands with a similar diameter for each device and with a standard deviation of less than 10% of the average diameter, indicating the reproducibility of fabricating alginate hydrogel microstrands using these SiS devices.

Using the 1 mL/5 mL and 1 mL/10 mL SiS devices allowed us to fabricate microstrands with diameters of around 200 µm, generally believed to be the limit for adequate oxygen mass transfer [80], showcasing the potential for fabricating hydrogel-based cell-delivery vehicles for implantation studies using a syringe-based handheld SiS device. Compared to 1 mL/10 mL SiS devices, 1 mL/5 mL devices generated less negative pressure for microstrand formation, making the fabrication process better controlled. Therefore, we chose 1 mL/5 mL SiS devices for the subsequent studies. The morphology of microstrands made by 1 mL/5 mL SiS devices was determined by SEM, in which the air-dried sample showed a smooth surface with a compact network-like structure on the surface (Figure S3a), and the lyophilized sample showed a rough surface with internal porous structure (Figure S3b).

To further characterize the alginate hydrogel microstrands, we measured the mass swelling ratio and connective porosity of these microstrands in DMEM for 7 days. After fabrication, the swelling ratio of the microstrands was 22.7 ± 3.4% (day 0). After incubation in DMEM for 1 day, the swelling ratio significantly increased to 32.4 ± 1.6% (day 1 compared to day 0, *p* < 0.01) and then stayed at 26–27% on days 4 and 7 (Figure 1f). The average of the swelling ratio on days 0, 1, 4, and 7 was 27.0 ± 4.0%, indicating that these alginate hydrogel microstrands fabricated by the SiS device might have relatively dense crosslinking and relatively constant swelling behavior with time. The connective porosity exhibited a similar trend; i.e., the porosity did not show significant changes during the 7-day incubation with DMEM (Figure 1g). The average of the connective porosity measured on days 0, 1, 4, and 7 was 56.1 ± 4.2%.

### 3.2. Cell Encapsulation and Recovery Efficiency from Alginate Hydrogel Microstrands

**Figure 2:**
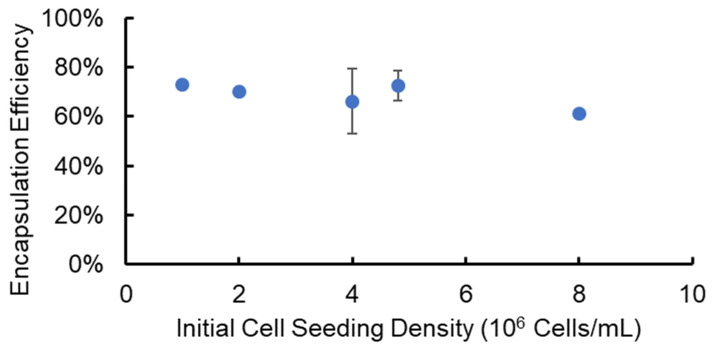
Efficiency of cell encapsulation and recovery from alginate hydrogel microstrands containing NIH 3T3 fibroblasts at different initial cell seeding densities.

To determine the effectiveness of using the SiS device for cell delivery and implantation research, we encapsulated cells in alginate hydrogel microstrands with a known cell number, released the cells from the microstrands after a three-hour incubation, and determined the cell encapsulation and recovery efficiencies. Specifically, we utilized NIH 3T3 fibroblasts as a stromal cell model at varying initial cell seeding densities (1–8 × 10^6^ cells/mL alginate solution) and encapsulated cells in alginate hydrogel microstrands using the 1 mL/5 mL SiS device. Following a three-hour incubation period post-fabrication, these cell-laden microstrands were dissolved using 55 mM sodium citrate solution to release the cells. The total cell number and viable cell number recovered from each set of alginate hydrogel microstrands were measured using a trypan blue exclusion assay. We calculated the encapsulation and recovery efficiency by dividing the total cell number after release from each set of microstrands by the initial cell number prior to encapsulation. The encapsulation and recovery efficiency averaged 69 ± 6% across different initial cell seeding densities, ranging from 1 to 8 × 10^6^ cells/mL alginate solution (Figure 2).

### 3.3. Evaluation of Cell Viability in Alginate Hydrogel Microstrands

**Figure 3:**
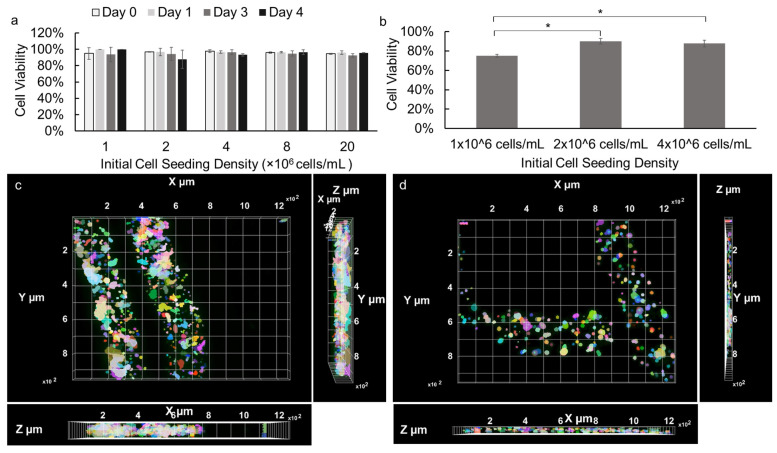
Viability of cells cultured in alginate hydrogel microstrands. (**a**) NIH 3T3 fibroblasts encapsulated in microstrands at various initial cell seeding densities (1–20 × 10^6^ cells/mL alginate), cultured for 0, 1, 3, and 4 days. (**b**) Primary E16 mesenchyme cells encapsulated in microstrands at various cell seeding densities (1–4 × 10^6^ cells/mL alginate) cultured for 3 days. * *p* < 0.05. (**c**) Top-view of 3D-reconstructed fluorescence image of LIVE/DEAD stained NIH 3T3-laden microstrands at cell seeding density 2 × 10^7^ cells/mL on day 4. Bottom, X-Z view. Right, Y-Z view. (**d**) Top, view of 3D reconstructed fluorescence image of LIVE/DEAD stained E16-laden microstrands at cell seeding density 4 × 10^6^ cells/mL on day 4. Bottom, X-Z view. Right, Y-Z view. Pseudo-colors were used to visualize individual cells or cell aggregates.

The cell viability of NIH 3T3 fibroblasts and primary E16 mesenchyme cells encapsulated in alginate hydrogel microstrands were assessed during 4- and 3-day growth periods, respectively. NIH 3T3 fibroblasts (1 × 10^6^ cells) were encapsulated in 250 µL alginate hydrogel microstrands and cultured for 0, 1, 3, or 4 days, with the culture medium being changed daily. As shown in Figure S4, cell viability of NIH 3T3 fibroblasts in microstrands remained ≥90% for four days. Next, the effect of initial cell seeding density on cell viability during high-density cell culture in microstrands was further evaluated. Our results demonstrated that over 90% of NIH 3T3 fibroblasts remained viable when encapsulated in the alginate hydrogel microstrands at various initial cell seeding densities ranging from 1 to 20 × 10^6^ cells/mL alginate and cultured for 0, 1, 3, and 4 days (Figure 3a). The viability of NIH 3T3 fibroblasts in alginate hydrogel microstrands at various initial seeding densities was further confirmed by LIVE/DEAD assay, revealing that the majority of cells within the microstrands remained viable on day 4 (Figure S5). Similarly, primary E16 mesenchyme cells at initial cell seeding densities ranging from 1 to 4 × 10^6^ cells/mL alginate were encapsulated in microstrands. While 1 × 10^6^ cells/mL alginate encapsulated microstrands exhibited 75% cell viability, 2 × 10^6^ cells/mL alginate and 4 × 10^6^ cells/mL alginate encapsulated microstrands exhibited over 90% cell viability on day 3 (Figure 3b). In addition, the LIVE/DEAD assay revealed that the majority of primary E16 mesenchyme cells within the microstrands at an initial seeding density of 4 × 10^6^ cells/mL alginate remained viable on day 4 (Figure S6). From both optical images and fluorescence images of NIH 3T3 fibroblasts (Figure S5) and E16 mesenchyme cells (Figure S6), we observed that cells were evenly distributed and organized into small cell aggregates throughout the microstrands presented. We further reconstructed Z stack images of LIVE/DEAD stained NIH 3T3 fibroblasts at an initial seeding density of 1 × 10^7^ cells/mL microstrands on day 4 (Movie S1) and primary E16 mesenchyme at 4 × 10^6^ cells/mL microstrands on day 4 (Movie S2), confirming that cells exhibited relatively uniform distribution in the 3D space of these alginate hydrogel microstrands. The X-Y, Y-Z, and X-Z views of NIH 3T3-laden microstrands (Figure 3c) and E16-laden microstrands clearly showed that cells were evenly distributed throughout the segment of the alginate hydrogel microstrand being imaged; cells at higher seeding density (1 × 10^7^ cells/mL, Figure 3c) formed relatively larger and more aggregates in alginate hydrogel microstrands when compared to lower seeding density (4 × 10^6^ cells/mL Figure 3d).

### 3.4. Assessment of Cell Growth in Alginate Hydrogel Microstrands

**Figure 4:**
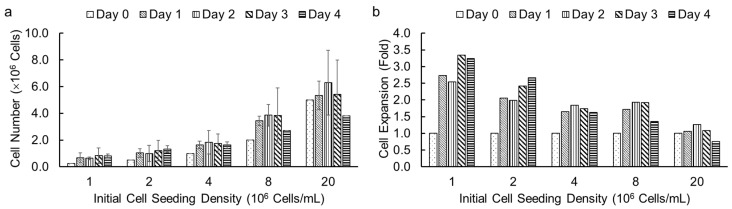
Cell growth of NIH 3T3 fibroblasts in 250 µL alginate hydrogel microstrands determined by alamarBlue assay at different initial cell seeding densities (1–20 × 10^6^ cells/mL alginate) for 4 days. (**a**) Viable cell number. (**b**) Cell expansion in fold change.

Following the successful demonstration of high cell viability in alginate hydrogel microstrands for different cell types, we investigated the effect of initial cell seeding densities and culture duration on cell growth in microstrands using an alamarBlue assay. We encapsulated NIH 3T3 fibroblasts in alginate hydrogel microstrands at different initial cell seeding densities, ranging from 1 to 20 × 10^6^ cells/mL alginate solution, and determined viable cell number on days 0, 1, 2, 3, and 4, respectively. We chose the alamarBlue assay to measure cell number in microstrands each day because alamarBlue allows for non-invasive, real-time monitoring of viable cell number. The effect of initial cell seeding densities (1, 2, 4, 8, and 20 × 10^6^ cells/mL alginate) on cell growth for NIH 3T3 fibroblasts in microstrands is shown in Figure 4a. At initial cell seeding densities of 1–8 × 10^6^ cells/mL alginate, the cell-laden microstrands showed an increase in cell number on day 1 and then reached a plateau for 4 days. For an initial cell seeding density of 2 × 10^7^ cells/mL alginate solution, the cell-laden microstrands exhibited maintenance of cell number for three days (Figure 4a). For initial cell seeding densities of 8 × 10^6^ and 2 × 10^7^ cells/mL alginate, the cell number decreased on day 4 compared to day 3, although the difference was not significant (Figure 4a). The lower the initial cell seeding density, the greater the fold cell expansion (Figure 4b).

### 3.5. Culture of MSC-like Cells in Alginate Hydrogel Microstrands and Their Phenotype Maintenance

**Figure 5:**
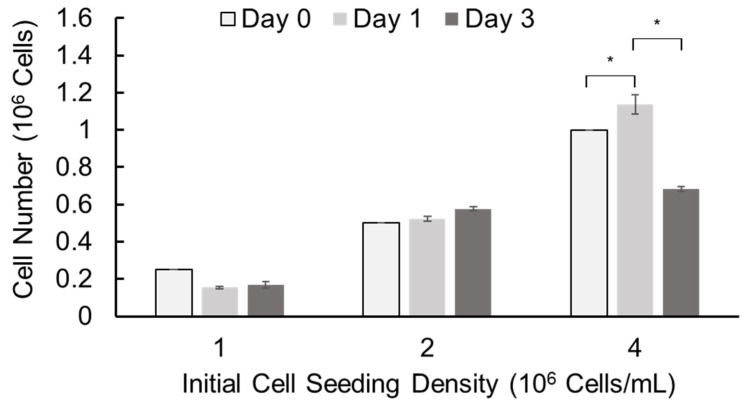
Cell growth of primary E16 mesenchyme cells in 250 µL alginate hydrogel microstrands determined by alamarBlue assay on days 0, 1, and 3. * *p* < 0.05.

We further assessed cell growth in alginate hydrogel microstrands by alamarBlue assay using murine E16 salivary mesenchyme cells as a primary stromal model. Primary E16 mesenchyme cells were encapsulated in alginate hydrogel microstrands at different initial cell seeding densities (1, 2, and 4 × 10^6^ cells/mL alginate) using the 1 mL/5 mL SiS device and cultured for 3 days (Figure 5). When the initial cell seeding density was 1 × 10^6^ cells/mL alginate solution, the cell number did not increase with culture duration. When the initial cell seeding density was 2 × 10^6^ cells/mL alginate solution, the cell number increased with the culture period. When the initial cell seeding density was 4 × 10^6^ cells/mL alginate solution, the cell number increased on day 1 but decreased on day 3. These data suggest that we might encapsulate primary stromal cells at 2 × 10^6^ cells/mL alginate solution and culture these cell-laden microstrands for up to 3 days or encapsulate cells at 4 × 10^6^ cells/mL alginate solution and culture for 1 day prior to cell implantation.

**Figure 6:**
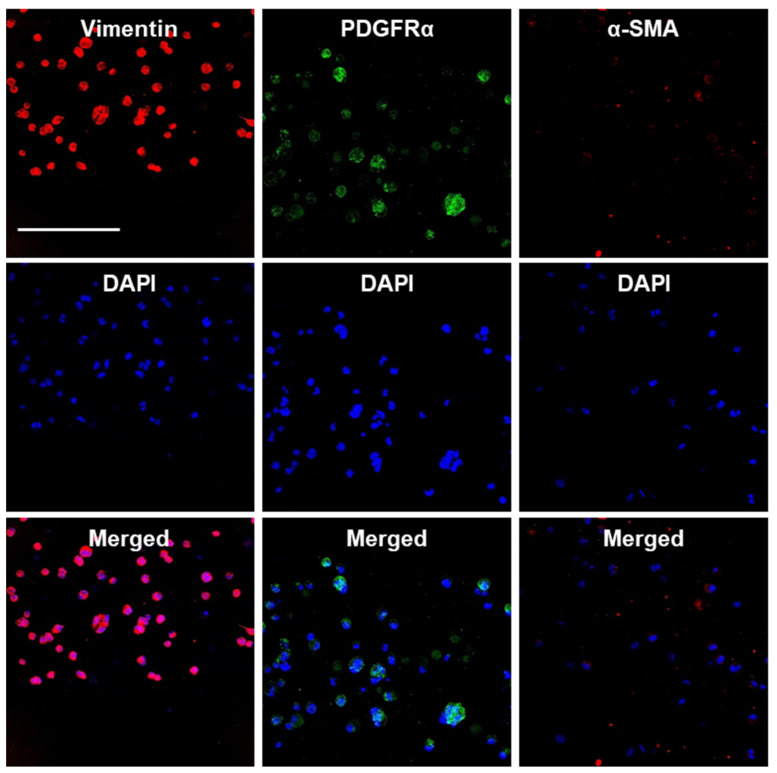
Confocal images of primary E16 mesenchyme cell cultured in microstrands showing expression of mesenchymal markers, vimentin (red) and PDGFRα (green), while showing minimal expression of the myofibroblast marker, α-SMA (red), on day 4. Middle panel: DAPI-stained cell nuclei in blue. Bottom panel: Merged images. Scale bar = 100 µm.

We also assessed the maintenance of the endogenous E16 stromal mesenchymal cell markers vimentin and PDGFRα and the myofibroblast fibrotic marker α-SMA in alginate hydrogel microstrands. When 1 × 10^6^ primary E16 mesenchyme cells were grown in 250 µL alginate hydrogel microstrands (i.e., 4 × 10^6^ cells/mL alginate solution) for 4 days, more than 96% of primary E16 mesenchyme cells maintained expression of native stromal mesenchymal markers, vimentin and PDGFRα, with minimal expression of α-SMA (Figure 6 and Figure S7). These results suggest that alginate hydrogel microstrands supported viable cell growth and retention of homeostatic mesenchymal phenotype without a conversion to a myofibroblast-like phenotype.

## 4. Discussion

Successful MSC-based therapies may benefit from effective delivery and retention of cells at the target site, which necessitates the development of suitable delivery vehicles [81,82]. This work strategically dealt with the fabrication and evaluation of alginate hydrogel microstrands for cell survival, growth, and phenotype maintenance for their potential use as cell delivery vehicles for MSCs.

Our invented, handheld SiS device provides a simple and straightforward approach to fabricating implantable, cell-laden alginate hydrogel microstrands or microfibers. Current major methods to fabricate alginate hydrogel microfibers include microfluidics [63,83,84,85,86], extrusion [66,67], and 3D bioprinting [87,88,89,90]. 3D bioprinting features precise control over the fabrication of intricate and complex structures by printing layer by layer, which cannot be attained by conventional fabrication strategies [91,92,93,94]. However, bioinks in 3D printers have limitations, including the requirement for pre-crosslinked bioink forms, the need for biocompatible formulations, the necessity for precise control over cell distribution and organization, and the optimization of bioinks for specific cellular applications [95]. Along with the long fabrication time, the bioink operation temperatures and the inability to handle high cell densities could cause damage to cells during 3D printing [96,97,98]. Clogging of needle tips is another significant problem faced in extrusion and extrusion-based bioprinting, particularly at high cell densities, which can cause cell over-accumulation both in the print head and small features [99,100,101,102]. Microfluidics are one of the most used methods for the fabrication of alginate hydrogel microfibers. However, significant time is required to learn the process and design a device to match/optimize the flow rates in each channel [103,104,105]. In our current approach to using the SiS device, the uniformly mixed cell–alginate solution is loaded in the alginate syringe, and 100 mM CaCl_2_ solution is loaded in the cross-linker syringe. By simply withdrawing the cross-linker syringe, a negative pressure is created that pulls the cell–alginate solution from the alginate syringe with the connecting needle into the cross-linker syringe. The negative pressure appears to prevent clogging of the needle tip. Using the SiS technique, we could easily and reproducibly fabricate alginate hydrogel microstrands with diameters of around 200 µm, whose swelling properties remained relatively constant during 7 days of incubation with DMEM, evidenced by their mass swelling ratio and connective porosity. In the future, the chemo-mechanical model [106,107] can be used to better understand the swelling behavior and mechanical properties of these alginate hydrogel microstrands.

Here, we have also demonstrated the ability of the SiS device to handle high cell densities of up to 20 million cells per mL alginate solution with an encapsulation and recovery efficiency of around 70%. The encapsulation efficiency could be potentially improved by utilizing zero-void syringes, which would be further optimized to minimize cell loss during encapsulation for cell delivery and implantation. The SiS device permits alginate hydrogel microstrand fabrication in a couple of minutes with high reproducibility. An ideal cell delivery vehicle provides a 3D environment for transplanted cells and enables them to maintain their viability and phenotypic characteristics, allowing them to restore the function of damaged or diseased tissues. 

In this work, the NIH 3T3 fibroblast-laden alginate hydrogel microstrands were fabricated with cell viability of around 95%, and such high cell viability was retained for 3 days of culture (Figure 3a), demonstrating higher cell viability compared to NIH 3T3 fibroblast-laden hydrogels fabricated by bioprinting [108] (e.g., 87% after printing, 85% after 48 h and 82% after 72 h [109]; 92.4% immediately after printing and 90.8% after 24 h [110]). These alginate hydrogel microstrands also support high cell viability and retention of endogenous cell phenotype markers in primary mesenchymal stromal cells; for example, more than 95% of primary E16 mesenchyme cells expressed stromal mesenchymal markers, vimentin and PDGFRα. Altogether, our results support the applicability of the handheld SiS device for generating consistent and controlled microstrands for various research applications, including cell delivery and implantation of cells in vivo for regenerative medicine approaches.

The development of xerostomia or dry mouth syndrome is often accompanied by hyposalivation and fibrosis, which can lead to permanent scarring and organ dysfunction. Despite extensive research, current pharmacological interventions have not been highly successful in treating xerostomia [111,112]. Stem cell-based therapies, particularly using MSCs, have shown promise in regenerating salivary gland function due to their regenerative, anti-fibrotic, and anti-inflammatory properties [113,114]. The usefulness of MSCs for salivary gland regeneration has been demonstrated by injection of MSCs into damaged salivary glands in humans [113] or through the tail vein in mouse models [104,113,114]. However, clinical application of MSCs requires high cell expansion in scaffold systems prior to transplantation [115,116], and in vivo inflammatory molecules or the environment have adverse effects on the immunogenicity, viability, and differentiation capacity of MSCs [117]. To counteract MSC clearance by the recipient immune system [118,119], retention and homing of transplanted MSCs are necessary. Other bioengineered approaches have been tried for salivary gland regeneration, including controlled release of drugs such as pilocarpine and cevimeline [111], gene therapy [120,121,122], and fabrication of different biomaterials [123,124]. These approaches are limited by adverse side effects, mutagenesis, and host tissue responses to the transferred biomaterials, respectively.

Alginate hydrogels have been used for MSC delivery and implantation for different organ regeneration studies, such as chondrogenesis [125], stomach wall regeneration [32], bone [126,127], and nerve regeneration [128]. Recently, alginate hydrogels have been used as drug-delivery vehicles for the regeneration of salivary glands. In particular, alginate hydrogel microstrands provide advantages of high cell density encapsulation, high cell viability, controlled cell alignment, enhanced cell–cell interactions, high surface-to-volume ratio, and ease in handling and manipulation. Most studies using alginate hydrogel microfibers for in vivo implantation incorporated ECM proteins or growth factors to support cell growth [39,58,59,84]. Our work showed the ability to support cell viability above 90% using alginate hydrogels alone, providing a simple alginate hydrogel system that could be used in future implantation studies. This study was limited to short culture periods (no more than 4 days) due to the need for quick surgical implantation of freshly isolated or short-period cultured mesenchymal stromal cells. Culturing cells for 1–3 days would give the surgeon a flexible surgery window and give cells the opportunity to acclimate to the hydrogel environment. In future studies for MSC differentiation and organoid maturation in hydrogels, longer culture periods will be evaluated.

## 5. Conclusions

In this work, we used a simple and straightforward SiS device and method to fabricate cell-laden alginate hydrogel microstrands at small volumes for cell implantation and regeneration studies in mouse models. Combining the structural advantages of microstrands with unmodified alginate hydrogels as a cell delivery vehicle with high cell-loading density, high cell viability, and maintenance of native stromal mesenchymal cell markers was achieved in vitro. Using a 3D alamarBlue assay, stromal cell growth in alginate hydrogel microstrands was confirmed, providing guidance on initial cell seeding density and culture period for future cell transplantation studies. The developed SiS device and alginate hydrogel microstrands have the potential for broad applications in cell delivery, tissue engineering, and regenerative medicine. Subsequent investigations could explore the long-term effects of cell implantation using alginate hydrogel microstrands, as well as evaluate the potential of combining the microstrands with other macromolecules or growth factors to promote tissue regeneration and functional restoration. In conclusion, our studies provide a foundation for further research and development of advanced cell-based therapies and cell implantation studies using hydrogel-based cell delivery systems in various tissue engineering applications.

## Figures and Tables

**Table 1:** Diameter of alginate hydrogel microstrands fabricated by the Syringe-in-Syringe devices.

Device No.	Alginate Syringe Capacity (mL)	Cross-Linker Syringe Capacity (mL)	Needle Gauge (G)	Diameter of Microstrands (µm) ^a,^***				
				Set 1	Set 2	Set 3	Set 4	Average
1	1	1	30	not feasible				
2	1	3	30	290.7 ± 13.7	271.0 ± 7.4	295.0 ± 4.4	310.4 ± 9.5	291.8 ± 16.2
3	1	5	30	253.5 ± 8.6	213.4 ± 7.4	208.4 ± 1.7	211.2 ± 4.7	221.6 ± 21.3
4	1	10	30	202.3 ± 3.1	189.0 ± 1.7	183.8 ± 5.2	187.6 ± 4.4	190.7 ± 8.1
5	3	3	30	not feasible				
6	3	5	30	not feasible				
7	3	10	30	309.0 ± 3.6	311.9 ± 1.5	299.7 ± 3.6	299.7 ± 7.8	303.8 ± 5.7
8	5	5	30	not feasible				
9	5	10	30	not feasible				

^a^ Mean ± standard deviation (*n* = 3). ***: *p* < 0.0001 between the average diameters of microstrands made by devices No. 2, 3, 4, and 7.

## Data Availability

The authors declare that all data supporting the findings of this study are available within this paper and its supplementary data.

## Supplementary Materials

The following supporting information can be downloaded at https://www.mdpi.com/article/10.3390/bioengineering11040375/s1, Figure S1: alamarBlue standard curve for NIH 3T3 fibroblasts in alginate hydrogel microstrands with different amounts of alamarBlue reagent. (a) Adding 120 µL alamarBlue dye to cell-containing microstrands in 6 mL medium (2%). (b) Adding 600 µL alamarBlue dye to cell-containing microstrands in 6 mL medium (10%). Figure S2: Validation of cell number of NIH 3T3 cells in alginate hydrogel microstrands determined by alamarBlue assay with cell counting by trypan blue exclusion assay. Figure S3: SEM images of alginate hydrogel microstrands. (a) Air-dried sample. (b) Lyophilized sample. Scale bar = 20 µm. Figure S4: Cell viability of NIH 3T3 fibroblasts in alginate hydrogel microstrands. 1 × 10^6^ cells encapsulated in 250 µL alginate microstrands and cultured for 4 days. Figure S5: Cell growth of NIH 3T3 fibroblasts in alginate hydrogel microstrands for 4 days. (a) Optical images on day 0, 1, 3, and 4. (b) Fluorescence image of LIVE/DEAD stained cells in microstrands on day 4. Green, live cells. Red, dead cells. Scale bar = 650 µm. Figure S6: Primary E16 mesenchyme cells grown in alginate hydrogel microstrands at an initial cell seeding density of 4 × 10^6^ cells/mL alginate for 4 days. (a) Optical image. (b) Fluorescence image of LIVE/DEAD stained cells in microstrands. Green, live cells. Red, dead cells. Scale bar = 275 µm. Figure S7: Quantification of confocal images of primary E16 mesenchyme cells cultured in microstrands showing more than 96% primary E16 mesenchyme cells expressing stromal mesenchymal markers, vimentin and PDGFRα, while less than 40% cells express the myofibroblast marker, α-SMA, on day 4. Movie S1: Cell distribution of 1 × 10^7^ NIH 3T3 fibroblasts per milliliter alginate hydrogel microstrands reconstructed from Z-stacked fluorescent images of LIVE/DEAD assay on day 4. Movie S2: Cell distribution of primary E16 mesenchyme cells per milliliter alginate hydrogel microstrands reconstructed from Z-stacked fluorescent images of LIVE/DEAD assay on day 4.
