# Supplementary material for: Evaluation of Alginate Hydrogel Microstrands for Stromal Cell Encapsulation and Maintenance
Source: Bioengineering (Basel). 2024 Apr 13;11(4):375. doi: 10.3390/bioengineering11040375 (PMC11048715; doi:10.3390/bioengineering11040375)
Supplement: Supplementary file 1 [file bioengineering-11-00375-s001.zip › bioengineering-2940875-supplementary.pdf]

Evaluation of Alginate Hydrogel Microstrands for Stromal Cell Encapsulation and Maintenance

Supplementary Materials

Scanning electron microscopy (SEM) of alginate hydrogel microstrands

Alginate hydrogel microstrands were either air dried at room temperature overnight or vacuum-dried using the FreeZone Plus 2.5L -84C Cascade Benchtop Freeze Dryer System (Labconco, Kansas City, MO, USA) at -83°C under 6 Pa overnight. Samples were mounted onto SEM specimen stubs with carbon tape and sputter coated with iridium-palladium. SEM images were taken using a Zeiss Leo 1550 field emission scanning electron microscope (Zeiss Leo Electron Microscopy Ltd., Cambridge, UK) at 3.00 kV.

Supplemental Figure S1–S7

Supplemental Movie S1. Cell distribution of 1 × 10^7 NIH 3T3 fibroblasts per milliliter alginate hydrogel microstrands reconstructed from Z-stacked fluorescent images of LIVE/DEAD assay on day 4.

Supplemental Movie S2. Cell distribution of primary E16 mesenchyme cells per milliliter alginate hydrogel microstrands reconstructed from Z-stacked fluorescent images of LIVE/DEAD assay on day 4.

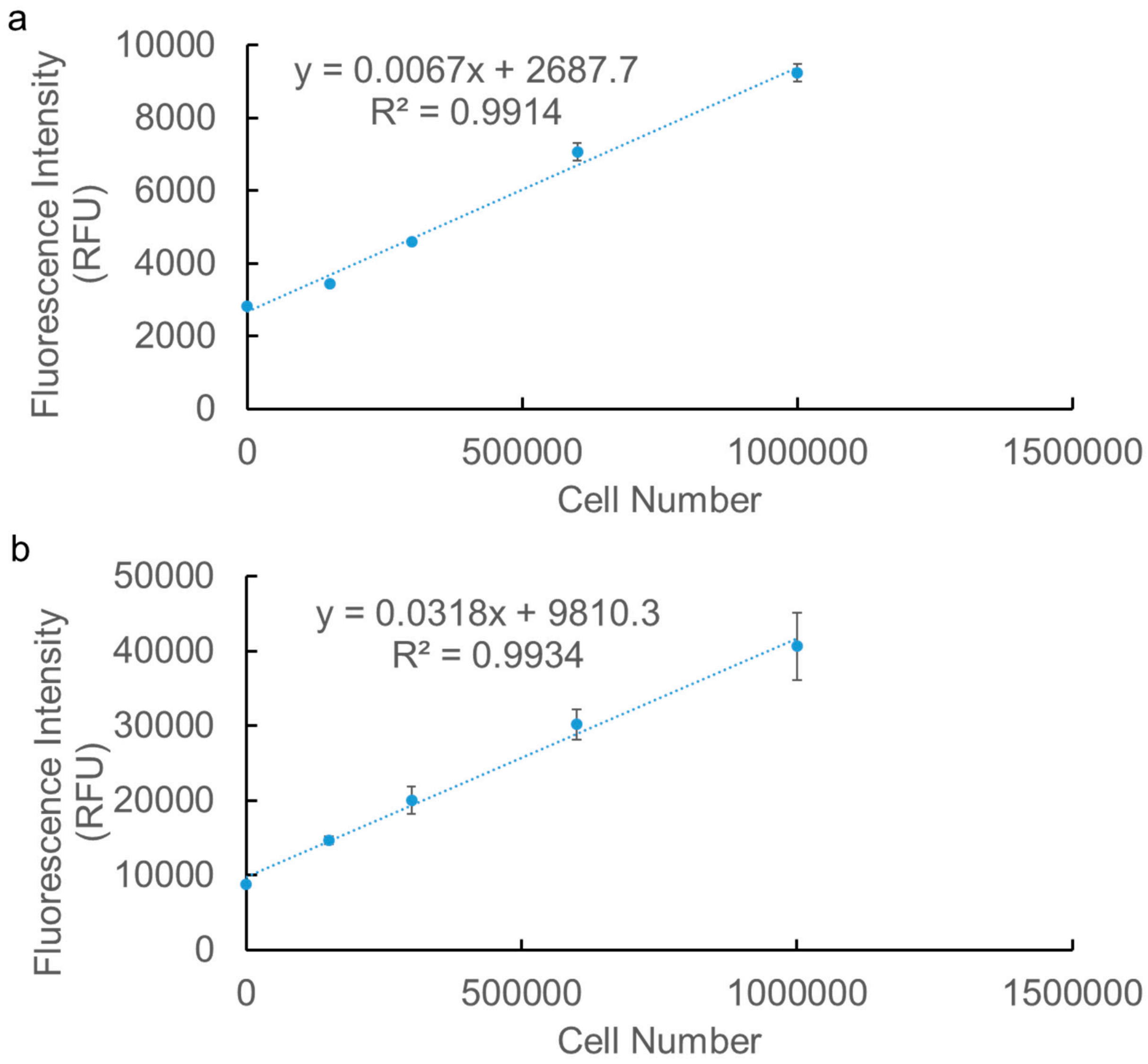

Figure S1. alamarBlue standard curve for NIH 3T3 fibroblasts in alginate hydrogel microstrands with different amounts of alamarBlue reagent. (a) Adding 120 μL alamarBlue dye to cell-containing microstrands in 6 mL medium (2%). (b) Adding 600 μL alamarBlue dye to cell-containing microstrands in 6 mL medium (10%).

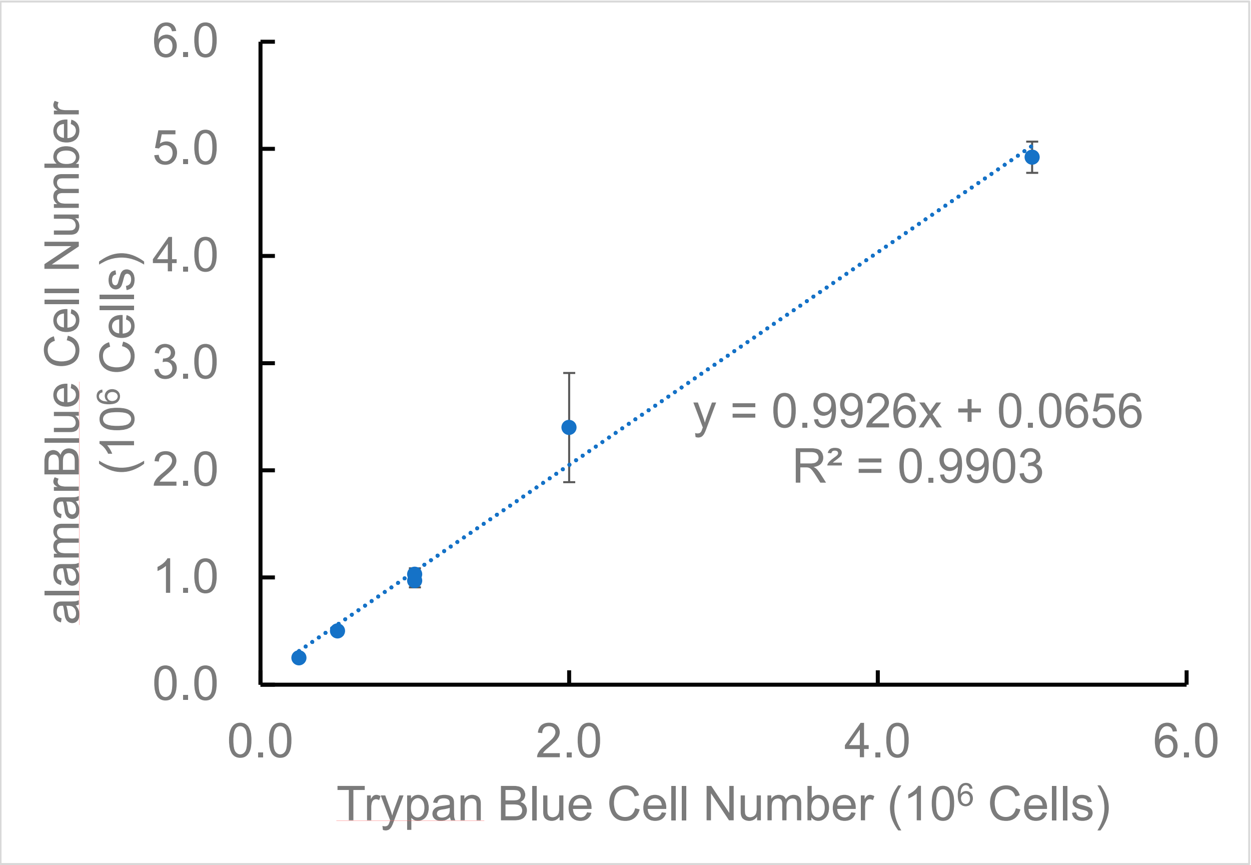

Figure S2. Validation of cell number of NIH 3T3 cells in alginate hydrogel microstrands determined by alamarBlue assay with cell counting by trypan blue exclusion assay.

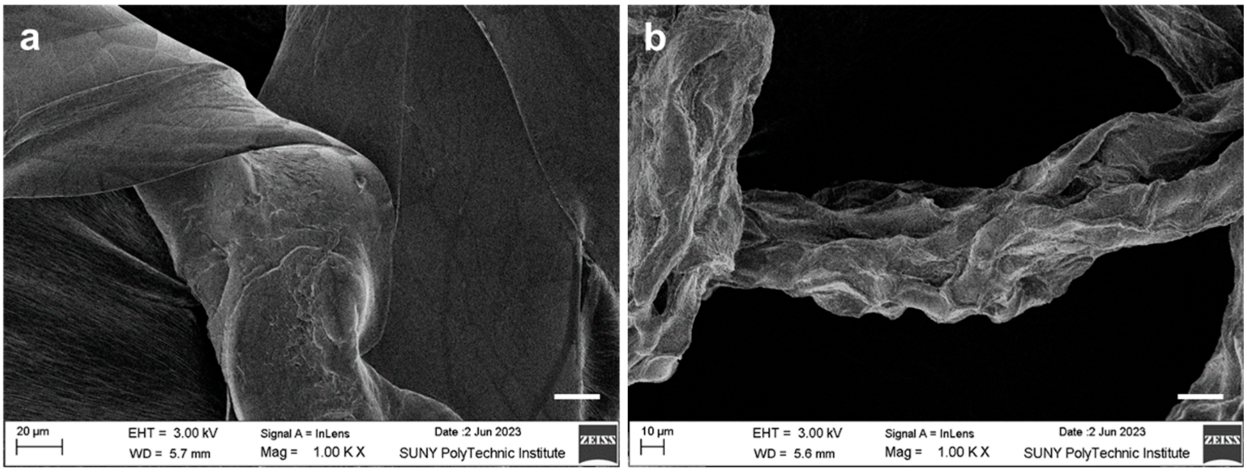

Figure S3. SEM images of alginate hydrogel microstrands. (a) Air-dried sample. (b) Lyophilized sample. Scale bar = 20 μm.

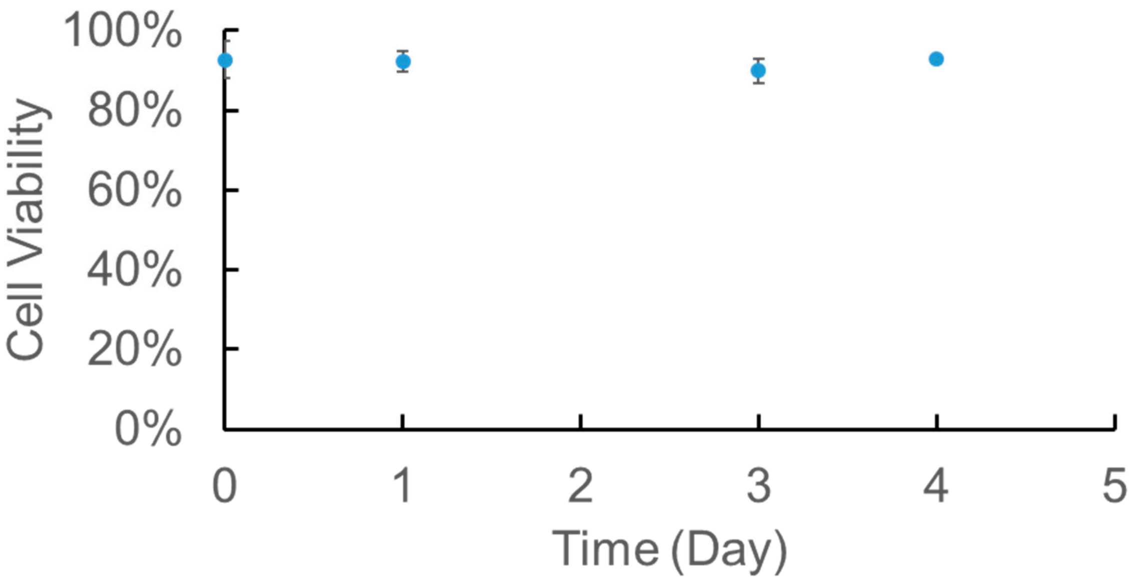

Figure S4. Cell viability of NIH 3T3 fibroblasts in alginate hydrogel microstrands. 1×10^6 cells encapsulated in 250 μL alginate microstrands and cultured for 4 days.

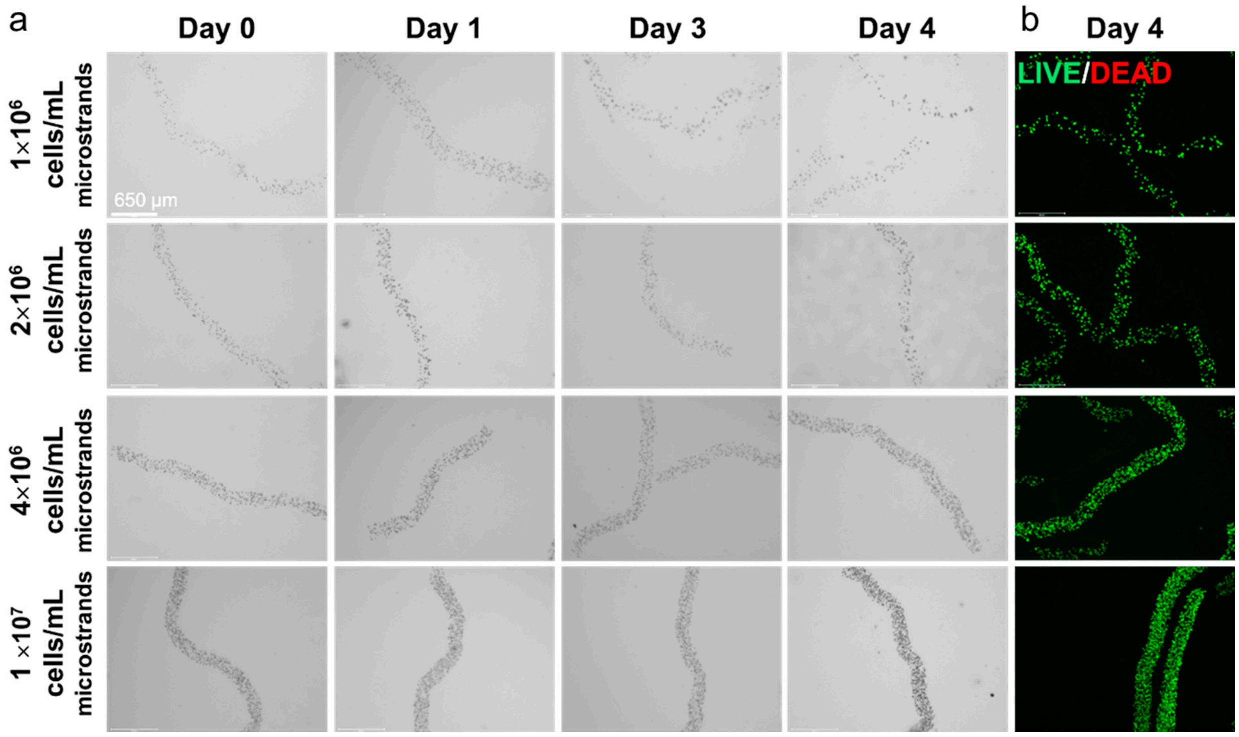

Figure S5. Cell growth of NIH 3T3 fibroblasts in alginate hydrogel microstrands for 4 days. (a) Optical images on day 0, 1, 3 and 4. (b) Fluorescence image of LIVE/DEAD stained cells in microstrands on day 4. Green, live cells. Red, dead cells. Scale bar = 650 μm.

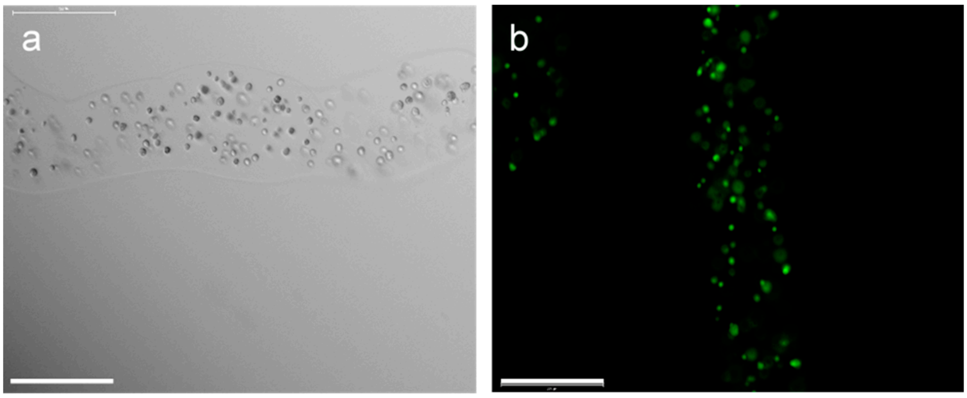

Figure S6. Primary E16 mesenchyme cells grown in alginate hydrogel microstrands at an initial cell seeding density of 4×10^6 cells/mL alginate for 4 days. (a) Optical image. (b) Fluorescence image of LIVE/DEAD stained cells in microstrands. Green, live cells. Red, dead cells. Scale bar = 275 μm.

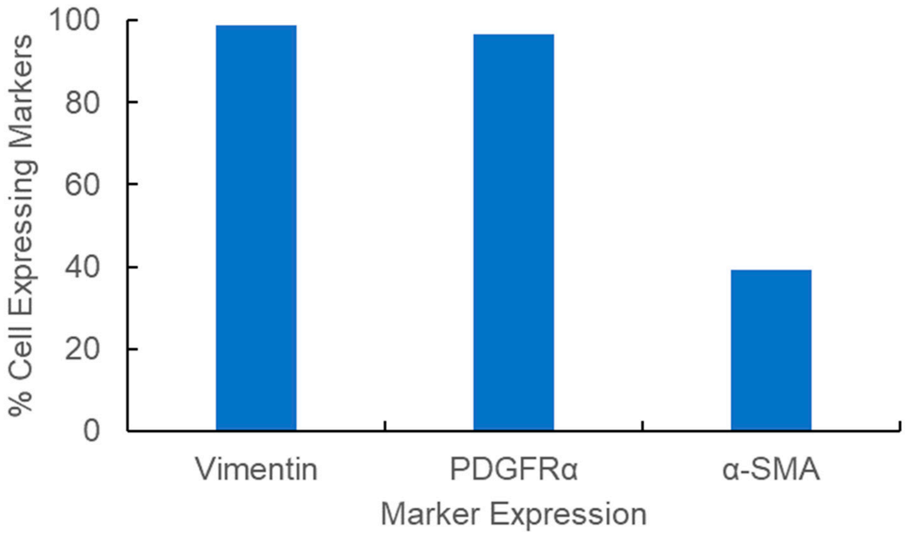

Figure S7. Quantification of confocal images of primary E16 mesenchyme cells cultured in microstrands showing more than 96% primary E16 mesenchyme cells express stromal mesenchymal markers, vimentin and PDGFRα while less than 40% cells expressing the myofibroblast marker, α-SMA on day 4.
